# Inflammation, Vasospasm, and Brain Injury after Subarachnoid Hemorrhage

**DOI:** 10.1155/2014/384342

**Published:** 2014-07-03

**Authors:** Brandon A. Miller, Nefize Turan, Monica Chau, Gustavo Pradilla

**Affiliations:** ^1^Department of Neurological Surgery, Emory University School of Medicine, Atlanta, GA, USA; ^2^Division of Neuropathology, Department of Pathology, Emory University School of Medicine, Atlanta, GA, USA; ^3^Cerebrovascular Research Laboratory, Grady Memorial Hospital, Emory University School of Medicine, 1365 Clifton Road, NE, Suite B6166, Atlanta, GA, USA

## Abstract

Subarachnoid hemorrhage (SAH) can lead to devastating neurological outcomes, and there are few pharmacologic treatments available for treating this condition. Both animal and human studies provide evidence of inflammation being a driving force behind the pathology of SAH, leading to both direct brain injury and vasospasm, which in turn leads to ischemic brain injury. Several inflammatory mediators that are elevated after SAH have been studied in detail. While there is promising data indicating that blocking these factors might benefit patients after SAH, there has been little success in clinical trials. One of the key factors that complicates clinical trials of SAH is the variability of the initial injury and subsequent inflammatory response. It is likely that both genetic and environmental factors contribute to the variability of patients' post-SAH inflammatory response and that this confounds trials of anti-inflammatory therapies. Additionally, systemic inflammation from other conditions that affect patients with SAH could contribute to brain injury and vasospasm after SAH. Continuing work on biomarkers of inflammation after SAH may lead to development of patient-specific anti-inflammatory therapies to improve outcome after SAH.

## 1. Introduction

Subarachnoid hemorrhage (SAH) remains a devastating disease, leaving survivors with neurological injuries that range from subtle cognitive deficits to disabling cerebral infarctions. While treatment continues to evolve and improve, there are few therapies that treat the underlying pathological mechanisms of SAH. Additionally, there is no clear explanation for the heterogeneity among patients with SAH, with some recovering well and others worsening after their initial ictus. In this review, we will discuss the evidence supporting the role of inflammation as a direct mediator of neurological injury after SAH and a causative factor of post-SAH vasospasm. We hypothesize that the diffuse inflammatory response after SAH results in acute and chronic neurological injury and vasospasm and that patients with more severe inflammatory responses may experience worse outcomes after SAH. An improved understanding of the inflammatory pathways activated after SAH will likely lead to novel therapies and improved patient outcomes.

## 2. Evidence for Acute Inflammation after Subarachnoid Hemorrhage

### 2.1. Detection of Inflammatory Mediators in CSF after SAH

Several human studies have repeatedly shown elevated inflammatory mediators within CSF after SAH. While the key mediators identified may vary across studies, the relationship between elevation, onset of vasospasm, and decreased neurological outcomes remains a consistent finding [1, 2]. A study by Polin and colleagues [3] showed that patients who developed vasospasm after SAH had higher CSF levels of E-selectin, an endothelial cell molecule that induces leukocyte adherence and extravasation and subsequent tissue injury in ischemic stroke [4–6]. These findings are supported by experimental data showing that CSF from patients with SAH increased rolling and adhesion of leukocytes in an* in vitro *mouse model [7]. While there are other studies showing elevation of E-selectin after SAH [8], some have failed to detect E-selectin in the CSF of patients with SAH, even when other inflammatory molecules, such as monocyte chemoattractant protein-1 (MCP-1), were elevated [9]. Other cytokines that have been widely cited to play a role in SAH, such as tumor necrosis factor-alpha (TNF*α*), also show wide variation in expression when compared across different studies. For instance, Kikuchi and colleagues found elevated levels of interleukin- (IL-) 6 and IL-8 but not TNF*α* after SAH, while other groups have found elevations of TNF*α* in the CSF after SAH [2, 10, 11]. One recent study found detectable levels of TNF*α* in only 30% of patients after SAH, indicating that the inflammatory response after SAH may be quite heterogeneous [12]. The differences across these studies may be the result of different CSF collection times after SAH, alternative methods of detection used, or diverse patient populations. In addition, cross contamination of CSF with blood during collection from ventricular or lumbar sources is rarely accounted for. The volume of blood present within the subarachnoid space would obviously affect the levels of cytokines present in CSF, and therefore cytokine concentration in CSF may reflect the volume of SAH rather than the magnitude of the inflammatory response within the brain.

One of the most widely studied molecules in SAH is endothelin-1 (ET-1), a vasoconstrictor produced by endothelial cells. ET-1 has been detected in CSF from patients with SAH and can be produced by monocytes isolated from CSF of SAH patients [13, 14]. ET-1 has been implicated in the development of vasospasm after SAH [15] and will be discussed in detail later in this review. As with many other proinflammatory molecules, the expression of ET-1 is highly variable: in a study by Fassbender and colleagues, ET-1 was not found in CSF of control subjects, and only 46% of patients with SAH had detectable levels of ET-1 [13]. Though the averaged results of both groups revealed a significant increase in ET-1 after SAH, this demonstrates that not all patients with SAH experience the same inflammatory response. Furthermore, a study from a different group failed to detect ET-1 after SAH [16]. This heterogeneity is readily apparent to clinicians treating SAH, as many patients move through their posthemorrhage course with few complications, while others experience severe complications such as vasospasm and cerebral edema, which may both be driven by an inflammatory response [17, 18].

### 2.2. Detection of Inflammatory Mediators in Blood after SAH

In addition to the inflammatory cytokines found within CSF in patients after SAH, a systemic increase in inflammatory mediators after SAH is well documented [1, 19, 20]. This systemic increase in inflammatory cytokines after SAH is predictive of poor outcome and may be related to a late, rather than early, inflammatory response [21–24]. Other markers of systemic inflammation, such as high body temperature and leukocytosis, have also correlated with worse outcomes after SAH; however, no causal relationship was established between peripheral inflammation and intracerebral pathology [25, 26]. There is increasing interest in developing inflammatory biomarkers for prognosticating outcome in SAH though no biomarker study in SAH has been prospectively validated [27]. It is unclear to what extent the presence of inflammatory cytokines in plasma is due to intracerebral processes or how strongly blood cytokine levels correlate with inflammation within the brain [12]. It is quite possible that inflammatory cytokines detected in plasma are indicators of global inflammation throughout the body, as would be expected in patients who are critically ill after SAH. For example, both ICAM-1 and E-selectin have been used to prognosticate outcome in critically ill patients* without* SAH [28]. It is also possible that inflammatory cytokines within the blood contribute to brain injury after SAH; the breakdown of the blood brain barrier after SAH could allow serum cytokines to enter the brain parenchyma leading to tissue injury there.

In planning any trial of anti-inflammatory therapy after SAH, consideration should be given to when the peak of inflammation occurs, in order to optimize delivery and dosing of the therapy. There is evidence that the expression of certain inflammatory molecules peaks within 24 hours after hemorrhage onset, with IL-6 being elevated at days 0 to 1 after SAH [29] though others have shown a peak of IL-6 at day 6 after SAH [30]. Once again, differences between these two studies may be due to many factors, including the high degree of variability among inflammatory responses among patients with SAH [3, 10].

### 2.3. Inflammation and Aneurysm Formation

Inflammation may also play a role in aneurysm formation. Several previously discussed inflammatory cytokines, such as TNF*α* and MCP-1, are thought to play a role in endothelial injury and remodeling based on evidence from both human and animal studies [31, 32]. A recent clinical study found increased cyclooxygenase within the walls of ruptured and unruptured aneurysms [33] and the same group has shown that aspirin may reduce the rate of aneurysm rupture due to its anti-inflammatory properties [34, 35]. E-selectin has also been found at elevated levels in the walls of aneurysms [36]. Additionally, environmental factors that contribute to a proinflammatory state may contribute to aneurysm formation [37]. A detailed discussion of the contribution of inflammation to aneurysm formation is outside the scope of this review and was reviewed recently elsewhere [38].

## 3. Evidence for Inflammation in Animal Models of SAH

Animal models have been utilized to establish a causative relationship between inflammation and brain injury after SAH. Several animal models for SAH exist, each with their own advantages and drawbacks [39]. Common models include blood injection into the basal cisterns of animals or endovascular perforation, both of which produce vasospasm and an inflammatory response [40–42]. As in human SAH studies, there is considerable variability between subjects when inflammatory responses are quantified [43, 44]. Animal studies of SAH have found evidence of inflammation in all intracerebral compartments: CSF, brain parenchyma, and vasculature [45–49].

Animal studies have been used to link inflammation, cerebral edema, and cell death after SAH. The anesthetic isoflurane has been shown to reduce TNF*α* production, leukocyte adhesion molecule expression, and blood brain barrier permeability after experimental SAH [50, 51]. The results of these studies are promising but do not prove whether decreased TNF*α* levels in affected tissue are a cause or result of decreased leukocyte infiltration. There is, however, evidence of TNF*α* having a direct role on neuronal injury after SAH. Recently, blockade of TNF*α* was shown to reduce apoptosis in the hippocampus after SAH [52]. This corresponds well to the extensive literature demonstrating a role for TNF*α* in neuronal apoptosis in other forms of neurological injury [53]. However, the antiapoptotic effects of TNF*α* blockade were not uniform throughout the brain, and a pre-rather than posttreatment paradigm was utilized in this study [52]. Furthermore, there is not even a consensus that neuronal death occurs in SAH, with some groups reporting no neuronal apoptosis after SAH while others observe neuronal death throughout the brain after SAH [54–56]. Regardless of whether or not neuronal death occurs after SAH, there are other pathological events that could explain neurological dysfunction after SAH, such as synaptic injury, loss of long term potentiation, and white matter injury [57]. Both synaptic loss and white matter injury are mediated by inflammation in models of other neurological diseases; however, more work is needed to understand the precise role of inflammation on cell death and injury after SAH [58, 59].

Animal models of SAH have also found evidence for inflammation playing a role in injury outside of the brain. In a study using endovascular perforation as a model of SAH in rats, systemic anti-inflammatory treatment was able to reduce lung injury after SAH [60]. This is relevant to SAH treatment as many patients experience cardiopulmonary complications as part of a systemic reaction to SAH [61]. Animal models of SAH have played an essential role in linking inflammation to vasospasm after SAH and will be discussed in more detail below.

## 4. The Role of Inflammation in Vasospasm after SAH

### 4.1. Induction of Vasospasm with Proinflammatory Agents

Early clinical studies showing a correlation between vasospasm and fever in the absence of infection established a link between inflammation and vasospasm [77–86]. Several proinflammatory agents such as talc (crystallized hydrous magnesium sulfate) [87, 88], latex, polystyrene, and dextran beads [89, 90], lipopolysaccharide (LPS) [91], and tenascin-C [92] have been administered intracisternally to show that vasospasm can occur in the absence of blood. These studies provided proof that vasospasm is not dependent on red blood cells (RBCs) or hemoglobin (Hgb) and confirmed the role of inflammation in the development of vasospasm.

### 4.2. Inflammatory Molecules Linked to Development of Vasospasm

Among inflammatory molecules linked to cerebral vasospasm, the selectin family, which consists of three members: E-selectin, platelet- (P-) selectin, and leukocyte- (L-) selectin, has been extensively studied. These molecules facilitate leukocyte binding and migration through vascular endothelium towards injured tissue. L-selectin and E-selectin are constitutively expressed on cell surfaces whereas P-selectin expression requires activation by histamine or thrombin [93]. E-selectin is elevated in the CSF of SAH patients with higher concentrations seen in patients who develop moderate or severe vasospasm [3]. Inhibition of E-selectin with an inhibitory antibody [62] and E-selectin tolerization via intranasal administration have decreased vasospasm in rodent SAH models [63]. However, not all data point to selectins having deleterious effects after SAH: while P-selectin levels were higher in patients with SAH who developed cerebral ischemia after SAH, L-selectins were higher in patients who did not develop delayed ischemia [23, 94].

Integrins are cell surface proteins that facilitate cell-cell adhesion and interaction. The main integrins involved in leukocyte adhesion and migration are lymphocyte function-associated antigen 1 (LFA-1) and Mac-1 integrin (CD11b/CD18). Systemically administered anti-LFA-1 and Mac-1 monoclonal antibodies reduce vasospasm in rat [64], rabbit [65], and primate [66] SAH models. Immunoglobulin superfamily proteins, such as ICAM-1, play a role in leukocyte adhesion and are upregulated in patients who develop clinical vasospasm [3] as well as in rabbit [70] and canine SAH models [47]. Anti-ICAM-1 monoclonal antibodies were shown to decrease femoral artery vasospasm and inhibit infiltration of macrophages and neutrophils into blood vessel adventitia in a rodent model [95] and reduce vasospasm in a rabbit model of SAH [71].

Key proinflammatory cytokines elevated in experimental models and patients with vasospasm include IL-1B, IL-6, IL-8, TNF*α*, and MCP-1. IL-6 has been shown to peak early after SAH, suggesting that it may be an early marker for predicting vasospasm development [9, 11, 47, 96–101]. TNF*α* levels in poor-grade SAH patients were shown to correlate with severity of vasospasm [68] and serum MCP-1 levels were associated with predicting negative outcome but not severity of vasospasm [21]. Cytokine inhibitor CNI-1493 [72], anti-IL-6 antibodies [102], anti-IL-1B antibodies [67], and TNF*α* inhibitors [103] have all been shown to attenuate vasospasm in animal models.

Several studies have examined intracellular signaling pathways activated during inflammation and their role in vasospasm. Mitogen-activated protein-kinase (MAPK) and nuclear factor kappa-B (Nf*κ*B) intracellular signaling pathways are crucial in generating inflammatory immune responses [104]. Jun N-terminal kinase 1 (JNK1) and JNK2, which are part of MAPK family, are activated in the cerebral vasculature after experimental SAH and their inhibition reduces vasospasm [105, 106]. Inhibition of JNK was also effective at reversing the vasoconstrictive effects of tenascin-C in a rat model of SAH [92]. Poly (ADP-ribose) polymerase (PARP) is a nuclear enzyme that regulates adhesion molecule expression and neutrophil recruitment during inflammation [107]. In a rabbit model of SAH, Satoh and colleagues showed PARP activation within the smooth muscle and adventitia of blood-exposed vessels and that PARP inhibition decreased the severity of vasospasm [108].

The complement pathway of antibacterial proteins also affects vasospasm after SAH. Complement depletion by treatment with cobra venom [109] and prevention of complement activation with nafamostat mesilate, a serine protease inhibitor, reduced vasospasm in experimental models [90, 110] and human subjects [111, 112]. Moreover, expression of the membrane attack complex (MAC) is increased in a rat model of SAH and can be responsible for lysis of extravasated erythrocytes and release of hemoglobin after SAH [113]. Recently, the lectin complement pathway (LCP) has also shown to be activated after SAH, and LCP activity has been linked to SAH severity and vasospasm in humans [114].

Oxidative signaling and oxidative stress are effectors of the immune response in many central nervous system diseases [115], and it is likely that the balance of oxidative stress and antioxidants influences response to and recovery from SAH. Haptoglobin is a serum protein composed of tetramer of two *α* and two *β* chains. Its main action is to bind free hemoglobin and facilitate its uptake and clearance. This has a net effect of reducing oxidative stress caused by free hemoglobin [116, 117]. Three phenotypes of haptoglobin (Hp) have been identified in humans: Hp 1-1, Hp 2-1, and Hp 2-2 [118]. In humans, the haptoglobin proteins with *α*-2 subunits have been associated with higher rates of vasospasm compared to haptoglobin *α*1-*α*1 [119]. Similarly, genetically modified Hp 2-2 mice experience increased macrophage infiltration in the subarachnoid space, more severe vasospasm, and worse functional outcome after SAH [120]. Recently, Hp 2-2 phenotype was associated with neurological deterioration but not cerebral infarction or unfavorable outcome in one clinical SAH study [121]; however, another recent study did find worse clinical outcomes in patients with the 2-2 phenotype [122]. Ongoing work in this area will further clarify the role of haptoglobin phenotype in SAH.

Endothelium-derived relaxing factor or nitric oxide (NO) is synthesized enzymatically by three main nitric oxide synthase (NOS) isoforms, endothelial (eNOS), neuronal (nNOS), and macrophage inducible NOS (iNOS) [123]. Under physiologic conditions, NO affects signaling pathways for vasodilation and cytoprotection among many others [123, 124]. The function of eNOS can be altered in many disease states such as atherosclerosis, hypertension, and diabetes mellitus, in which case eNOS starts to produce superoxide anion (O_2_^−^) instead of NO, an alteration defined as “eNOS uncoupling” [125]. Increased eNOS and iNOS expression were detected in mice after SAH, and this physiological response to SAH is decreased in proinflammatory Hp 2-2 transgenic mice compared with Hp 1-1 mice [126, 127]. In an animal model of SAH, simvastatin was shown to recouple eNOS and improve outcome after SAH [128]. On the other hand, genetic elimination of eNOS in knockout mice reduces the incidence of vasospasm and reduces oxidative stress as measured by superoxide production but has no effect on iNOS [129]. eNOS knockout mice also exhibit reduced Zn^2+^ release after SAH, which was associated with reduced microthrombi formation and neuronal degeneration in the same experiment.

Endothelins, which are potent vasoconstrictors and proinflammatory mediators expressed by vascular endothelial cells and vascular smooth muscle cells [130], are thought to contribute to tissue inflammation and cerebral edema. Several studies have documented increased ET-1 levels in SAH patients with symptomatic vasospasm and that the amount of blood within the cisterns correlated with the level of ET-1 in CSF [73, 76, 131]. However, other studies have failed to find significant elevation of ET-1 after SAH or a correlation between ET-1 levels and vasospasm [16, 73]. Inhibition of ET-1 by administration of anti-ET-1 monoclonal antibodies [132], anti-ET receptor antibodies [74, 133], ET activation enzyme inhibitors [134], and levosimendan (which antagonizes the ET receptor) [135] was effective in decreasing vasospasm in some [74, 133] but not all studies [135, 136]. Transgenic mice overexpressing ET-1 experienced more pronounced vasospasm and cerebral edema and an ET-A receptor antagonist decreased vasospasm and edema in the same study [137].

### 4.3. Trials of Anti-Inflammatory Agents for Treatment of Vasospasm

As subarachnoid blood is thought to generate much of the acute inflammation in SAH, faster clearance of subarachnoid clot could potentially improve outcomes after SAH. This theory has been tested, and intrathecal administration of thrombolytic agents has decreased vasospasm and improved outcomes in primates [138] and humans [139–143] after SAH. A meta-analysis showed a beneficial effect of thrombolytic treatment, with absolute risk reductions of 14.4%; however, the results of the analysis were limited due to predominance of nonrandomized studies [144]. In a recent clinical study by Yamamoto et al., cisternal irrigation therapy using tissue plasminogen activator in patients who underwent surgical clipping of ruptured intracranial aneurysms decreased serum inflammatory markers, reduced the incidence of ischemic lesions, and was associated with better neurological outcome [145]. Kim and colleagues recently demonstrated that cisternal irrigation with papaverine, a phosphodiesterase inhibitor and potent vasodilator, was similar in effectiveness compared to the thrombolytic urokinase, both of which decreased incidence of vasospasm [146].

Corticosteroids are potent anti-inflammatories and have been used in several human SAH trials. Experimental administration of high-dose methylprednisolone has been shown to reduce angiographic vasospasm and ameliorate arterial wall abnormalities in dog models [147–149]. Human clinical studies by Chyatte and colleagues [150] showed that high-dose methylprednisolone treatment improved neurological outcomes, reduced mortality, and delayed cerebral ischemia. A multicenter study of intravenous hydrocortisone administration after SAH showed improved mental status, speech, and motor function at 1 month after treatment [151]. However, another randomized controlled trial of hydrocortisone did not show any effect on neurological outcome after SAH [152]. On the other hand, hydrocortisone has been shown to support hypervolemic therapy by attenuating natriuresis [153], inducing hypervolemia, and improving the efficiency of hypervolemic therapy [154]. However, a large double blind randomized control trial demonstrated a beneficial effect of methylprednisolone in functional outcome one year after SAH but no effect on vasospasm [155]. These studies underscore the fact that outcomes in SAH are not solely dependent upon the development of vasospasm.

Nonsteroidal anti-inflammatory drugs (NSAIDs) also have potent anti-inflammatory properties, mediated in part by inhibition of cyclooxygenase expression, which reduces prostaglandin synthesis [156]. NSAID administration significantly reduced the severity of vasospasm in an animal model of SAH; however, the mode of NSAID treatment in this model, 30 minutes before and 3 hours after SAH, is not applicable to human trials [157]. Ibuprofen inhibits femoral artery vasospasm and decreases periadventitial monocytes and macrophages after, in a rodent model of SAH, and can increase cerebrovascular diameter in monkeys and rabbits [158–160]. Chyatte and colleagues also demonstrated that ibuprofen prevented ultrastructural changes in the cerebral vessel walls of dogs after blood injection [149]. The nonsteroidal anti-inflammatory drugs parecoxib and celecoxib have also shown promise as treatment options for vasospasm [161–163]. As these drugs are relatively safe and well studied in humans, they hold promise for clinically applicable treatment options for vasospasm.

Immunosuppressants such as cyclosporine cause T-cell dysfunction by inhibiting interleukin-2 (IL-2) transcription [164] and have been tested in experimental SAH with varied success [165, 166]. Clinical studies with cyclosporine are also varied, showing no beneficial effect of cyclosporine in the outcome patients with severe SAH [167], but showing improved outcome in patients who underwent early clipping after SAH when combined with nimodipine [168–170]. Tacrolimus (FK-506), a newer immunosuppressant, did not prevent vasoconstriction and lymphocytic infiltrations in experimental SAH models [171–173].

Statins are 3-hydroxy-3-methylglutaryl coenzyme A reductase inhibitors clinically used as cholesterol-reducing agents. Their ability to reduce the expression of proinflammatory cytokines and inhibit leukocyte integrins confers their potent anti-inflammatory activity [174, 175]. Preconditioning of rats with atorvastatin has been shown to reduce the level of ET-1 while attenuating vasospasm, which could be a mechanism of antivasospastic effects of statins after SAH [176]. Simvastatin treatment before or after SAH was also shown to attenuate cerebral vasospasm and neurological deficits, possibly via endothelial nitric oxide upregulation [177], and decrease perivascular granulocyte migration [40]. Several clinical studies have shown that statins decrease serum ICAM-1 levels in hypercholesterolemic patients [174, 175, 178–180], which could explain the experimental findings associated with decreased leukocyte migration. However, clinical studies with statins have yielded mixed results. While one study showed improved 14-day functional outcomes and cerebral vasospasm in patients receiving statins prior to their SAH [181], more recent studies [182, 183] did not find significant differences in the severity of angiographic or clinical vasospasm or neurologic outcomes of patients receiving statins after SAH. Another case-control study showed that oral atorvastatin treatment decreased vasospasm and cerebral ischemia but did not lead to significant functional improvement 1 year after SAH [184]. In a phase-II randomized controlled trial enrolling 80 patients with SAH, patients treated with oral pravastatin 72 hours after SAH had a 32% reduction in vasospasm incidence, and vasospasm-related neurologic deficits and mortality were decreased by 83% and 75%, respectively [185]. Subsequently, pravastatin was also effective at sustaining the improved neurological outcome at 6 months after the treatment [186]. A Cochrane review of clinical trials on statins after SAH concluded that, in the only clinical trial that met criteria, although simvastatin improved vasospasm, mortality, and functional outcome, these benefits were not statistically significant [187]. Currently, clinical trials including simvastatin in aneurysmal subarachnoid hemorrhage (STASH) trial are ongoing (http://clinicaltrials.gov/show/NCT00731627).

Nitric oxide (NO) depletion contributes to the pathogenesis of cerebral vasospasm after SAH [188, 189]. Therefore, several NO donors have been investigated for treatment of vasospasm. Intrathecal NO supplementation via controlled-released polymers was shown to prevent vasospasm in rat and rabbit models of SAH [190, 191] and delayed polymer implantation 24 or 48 hours after SAH has been shown to be still effective at ameliorating vasospasm [191]. Several other studies have also shown that selective intracerebral NO injection, [192] intraventricular NO injection [193], and systemic nitrite infusions can improve the severity or decrease the incidence of vasospasm in experimental and clinical studies [189]. Intravenous sodium nitrate (NaNO_2_) was also shown to reduce the degree of vasospasm and nitrite, nitrate, and S-nitrosothiols concentrations in CSF were found to be increased compared to controls in primate model of SAH [194]. L-citrulline is an amino acid that when converted to L-arginine increases nitric oxide (NO) production by NO synthase (NOS), leading to vasodilation [195]. L-citrulline administration has been shown to prevent posthemorrhagic cerebral vasospasm in the transgenic Hp 2-2 model of SAH, improve neurological function as determined by PGA (posture, grooming, and ambulation) scores, and reverse the decrease in upregulation of iNOS and eNOS expression in Hp 2-2 animals compared with baseline levels in mice [126]. Besides vasodilation, NO supplementation can have anti-inflammatory effects through modulating leukocyte-endothelial cell interactions in the acute inflammatory response. Inhibitors of NO production increase leukocyte adherence [196], and NO modulates oxidative stress [197] and microvascular permeability [198, 199]. The anti-inflammatory effects of NO through prevention of leukocyte adhesion have been linked with its ability to inactivate the superoxide anion [200]. Besides ameliorating vasospasm, whether NO donors including citrulline can help recoupling of eNOS, decrease the inflammatory infiltration, and decrease neuronal apoptosis requires further investigation. Other NO donors such as sodium nitroprusside and nitroglycerin are not considered as potential candidates due to their side effects such as dose-limiting hypotension, cyanide toxicity, and tolerance development [201].

Clazosentan, a synthetic endothelin receptor antagonist (ETRA), has been investigated as a potential treatment for vasospasm after subarachnoid hemorrhage [202]. In the CONSCIOUS-1 trial, an intravenous infusion of clazosentan 5 mg/h decreased vasospasm but the study was not powered to detect changes in morbidity and mortality [203]. In CONSCIOUS-2, a phase-III randomized controlled trial, including 1,157 patients, clazosentan infusion up to 14 days after hemorrhage did not reduce vasospasm-related morbidity and mortality or improve functional outcome [75]. A meta-analysis of randomized controlled trials for ETRAs for the treatment of vasospasm, including 5 trials with 2601 patients, showed that ETRAs decreased incidence of angiographic vasospasm; however, they did not improve functional outcome, vasospasm-related cerebral infarction, or mortality [204]. As a result, the use of ETRAs in patients with SAH was not proven to be beneficial [204]. These studies reinforce that vasospasm alone cannot be accounted for the neurological deficits and functional outcome after SAH and treatment strategies that only target improving or preventing vasospasm are not likely to succeed.

Cilostazol is a selective phosphodiesterase III inhibitor that is used to treat ischemic peripheral vascular disease and exhibits anti-inflammatory properties including inhibiting microglial activation [205, 206]. Oral cilostazol administration prevented vasospasm in a rat model of SAH [207] and reduced endothelial damage in a canine model of SAH [208]. Clinical studies have demonstrated effectiveness of cilostazol in decreasing incidence and severity of vasospasm [209, 210]. A multicenter randomized clinical trial of cilostazol has shown a decrease in angiographic vasospasm but no improvement in outcomes 6 months after SAH [211].

## 5. The Role of Inflammation in Early Brain Injury and Delayed Neurological Deterioration after SAH

While there is no doubt that inflammation occurs after SAH, a link must be made between inflammation and poor outcomes after SAH for it to be considered an important therapeutic target. As demonstrated by several of the trials discussed above, vasospasm is not the only determinant of outcome after SAH. It is likely that inflammation plays multiple roles after SAH, mediating vasospasm as tissue damage as well as leading to regeneration or recovery as has been shown in other neurological conditions [212]. Alternately, the inflammatory cytokines detected in the CSF and blood after SAH could be results, rather than causative factors, of brain injury after SAH. There is a vast literature in ischemic stroke that ties the inflammatory response to deleterious effects such as edema and neuronal loss. Many experimental studies in stroke have shown blood brain barrier breakdown to be mediated by inflammatory cytokines [213] and that inhibiting inflammation reduces cerebral edema and neurological injury [214, 215]. Proinflammatory cytokines such as TNF*α*, IL-1, IL-6, and leukocyte adhesion molecules have been associated with worse outcomes in ischemic stroke [216]. However, despite the vast literature on inflammation in ischemic stroke, there have been no successful clinical trials using anti-inflammatory agents.

Recently, the term “early brain injury” has been used to describe the mechanisms of acute neurologic deterioration after SAH [217]. Early brain injury includes cell death, cerebral edema, and neuronal dysfunction that occur after SAH. Although vasospasm is a major cause of clinical deterioration after SAH, recent thinking has focused on the fact that vasospasm is not the only phenomenon contributing to poor patient outcomes after SAH [218, 219]. A key example of this is that nimodipine, the only pharmaceutical treatment shown to improve outcomes in SAH, appears to manifest its effects without affecting the rate of vasospasm [220, 221]. Many patients also undergo neurological deterioration in a delayed fashion after SAH. This can be due to cerebral vasospasm, which typically peaks 7 to 14 days after SAH, but also occurs in the absence of vasospasm. This subacute neurological decline is referred to as “delayed neurological deterioration” and can be mediated by several events including delayed ischemia (with or without vasospasm), seizures, and fevers [222]. The terms “delayed neurological deterioration (DND)” and “delayed cerebral ischemia (DCI)” are often used interchangeably, but it should be noted that DCI is just one of the causes of DND [223]. Additionally, it is likely that the events of early brain injury occur along a continuum with DND and are likely mediated by many of the same factors.

As in ischemic stroke, there is no accepted use of anti-inflammatory treatments for improving outcome after SAH. As discussed earlier, corticosteroids have been used to block inflammation after SAH, but there is no clear therapeutic benefit of this strategy [224]. However, there is experimental evidence from animal studies showing that blocking inflammatory pathways can improve both blood brain barrier breakdown and neuronal survival after SAH [219, 225]. In one study utilizing a rat model of SAH, there was a reduction in TNF*α* production, cerebral edema, and apoptosis in response to a blockade of a sulfonylurea receptor that is upregulated after SAH [45].

Clinically, the presence of cell death, cerebral edema, and vasospasm all contribute to poor outcomes after SAH. Though it is difficult to measure cell loss in humans, there is evidence for hippocampal neuronal loss after SAH based on MRI imaging, and elevated neurofilament levels in CSF correlate with functional outcome after SAH, indicating a link between axonal breakdown and clinical outcome [226, 227]. As in ischemic stroke, proinflammatory cytokines have gained attention as biomarkers for outcome in SAH, and the recent literature is well reviewed by Lad and colleagues [228]. Genetic differences in inflammatory cytokine expression and haptoglobin phenotype (discussed previously) have also been used to prognosticate outcome in patients with SAH, without definitive results [69]. Human genetic studies have also shown a benefit of lower TNF*α* levels for recovery after SAH [229]. This points to a possible dual role for inflammation in both acute injury and recovery, as has been proposed in other neurological diseases [230]. There is already experimental support for this dual role of inflammation in SAH, as the cytokine MCP-1 that has been associated with poor outcomes and vasospasm after SAH has recently been used to promote repair of experimental aneurysms [21, 231].

## 6. Discussion

**Figure 1:**
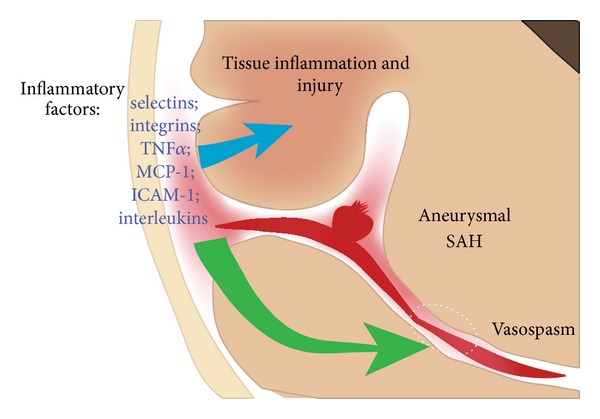
Schematic of a coronal projection of a ruptured cerebral aneurysm and contributing factors that result in cerebral vasospasm after SAH and delayed ischemic injury. Many inflammatory factors are hypothesized to contribute to brain injury and vasospasm after SAH. The interface between subarachnoid blood, brain parenchyma, and the cerebral vasculature is the likely location for induction of inflammatory cascades that lead to brain injury and vasospasm after SAH.

Evidence from both clinical and animal studies indicates that inflammation contributes to aneurysm formation, brain injury, and vasospasm after SAH and that many of the same molecules contribute to vasospasm and brain injury after SAH (Figure 1, Table 1). Much of the data from human studies linking inflammation to worse outcomes after SAH is correlative and studies examining different inflammatory molecules at different time points after SAH make it difficult to make direct comparisons (Table 1). However, taken together, human and animal studies suggest that a higher “inflammatory burden” contributes to the pathophysiology of SAH. This would suggest anti-inflammatory treatment to be a robust treatment strategy for SAH, as in other diseases. For example, the possibility that aspirin could reduce chronic inflammation within the walls of aneurysms and decrease the risk of rupture [34, 35] is akin to the paradigm of human cardiovascular disease in which the anti-inflammatory actions of aspirin and statins may protect against cardiovascular disease [122, 232, 233]. Unfortunately, this strategy has not borne out reliably in clinical trials. One potential reason for this is that animal studies of homogenous populations may not be an accurate model of SAH in humans where individual responses to a given insult could be quite variable.

Clinicians who care for patients with SAH understand that there is a wide range of physiologic responses to SAH, even in patients who present with the same initial grade of hemorrhage. While this is doubtlessly influenced by many factors (such as SAH blood volume), the intensity of an individual's inflammatory response to SAH may also determine if a patient develops delayed clinical deterioration or vasospasm. While this could be influenced by factors such as haptoglobin genotype [122], there are probably other genetic and environmental factors that influence patients' production of, and tolerance to, a post-SAH inflammatory response. Evidence from animal studies has shown that inflammatory stimuli can both exacerbate and reduce vasospasm, depending on the intensity of the stimulus [234]. A recent clinical study implied that preexisting atherosclerotic disease could have a protective effect on patients who suffer SAH, possibly by modifying neuroinflammation [233, 235, 236]. In the future, treatment for SAH may involve tailoring therapy to match the timing and intensity of an individual patient's inflammatory response. In order for this approach to be implemented, successful validation of inflammatory biomarkers and outcome measures for SAH would need to be developed.

## 7. Conclusion

The immune response within (and possibly outside of) the CNS is clearly a driving force behind many of the pathological events of SAH, including both vasospasm and early brain injury. Though much experimental and clinical work has linked increased inflammation to poor outcome after SAH, there is still no proven anti-inflammatory treatment that can be offered to patients who have suffered SAH. The volume of research on inflammation and SAH is rapidly expanding and will likely lead to new clinical trials, development of biomarkers, and hopefully anti-inflammatory treatments for SAH. Though anti-inflammatory treatments will likely improve the lives of patients with SAH, it must be remembered that neuroinflammation has beneficial effects as well and could also play a role in recovery after SAH.

## Figures and Tables

**Table 1:** Key inflammatory molecules implicated in the pathology of SAH.

Molecule	Function	Roles in animal studies	Roles in human studies	Comments
Selectins	Leukocyte adhesion	Inhibition of selectins decreases vasospasm [62, 63]	Higher levels in CSF correlate to vasospasm in some studies [3], found in walls of ruptured aneurysms [36]	Variable expression in patients with SAH [3, 8, 9], used to prognosticate outcome in critically ill patients without SAH [28]

Integrins	Leukocyte adhesion	Blocking reduces vasospasm [64–66]	Higher levels seen in patients with vasospasm [3]	

TNF*α*	Proinflammatory cytokine produced by leukocytes	Induces neuronal apoptosis after SAH [52]; blockade reduces vasospasm [67]	Found in CSF in patients after SAH and correlates with vasospasm after SAH [68]	Variable expression in patients with SAH [2, 10–12]

MCP-1	Macrophage chemoattractant	Promotes repair of aneurysms [69]	Found in CSF after SAH and associated with poor outcomes but not vasospasm [9, 21]	Also associated with vascular injury [31]

ICAM-1	Leukocyte adhesion	Increased in animal SAH studies [47, 70]; blockade reduces vasospasm [71]	Increased in patients with SAH [3]	Used to prognosticate outcome in critically ill patients without SAH [28]

Interleukins	Mediate leukocyte interactions	Blockade reduces vasospasm [72]	Peak early in SAH [9, 11]	Peak at variable times in human studies [29, 30]

Endothelin-1	Potent vasoconstrictor	Inhibition reduces vasospasm [73, 74]	Produced by monocytes from SAH patients [13, 14], with no proved benefit in clinical trials [75]	Highly variable expression after SAH and may not correlate with vasospasm [13, 16, 76]
